# Antithyroid autoantibody population as a source of antibody specificity and functionality: clinical significance of the phenomenon of the serological orchestra in monitoring of patients with Graves' disease

**DOI:** 10.1186/1878-5085-5-S1-A142

**Published:** 2014-02-11

**Authors:** Zemphira I Bogatyreva, Elena N Suchkova, Marina A Isaeva, Vladimir N Unanyan, Abner L Notkins, Sergey V Suchkov

**Affiliations:** 1 Division of Endocrinology, Municipal Clinical Hospital №81, Moscow, Russia; 2 AGMA and Alexandro-Mariinsky Clinical Hospital, Astrakhan, Russia; 3 University of World Politics and Law, Moscow, Russia; 4 I.M. Sechenov First MGMU, Moscow, Russia; 5 National Institutes of Health, Bethesda, MD, USA

## Introduction

The incidence and titers of serum anti-tissue and anti-organ autoantibodies (autoAT) depend on the clinical manifestations of autoimmune thyroid diseases and the expressiveness of the autoaggression. Among a family of anti-thyroid autoAbs the following ones should be highlighted:

(1) *canonical* (anti-TG and anti-TPO) autoAbs as well as combinatorial (*bispecific*: to TG and TPO simultaneously) autoAbs (so-called anti-TGPO autoAbs);

(2) autoAbs with the *functional* properties: anti-TSH-R autoAbs (TSH-R autoAbs), and autoAbs with proteolytic activity – *Ab-proteases*. Anti-thyroid autoAbs are occurring in patients with thyroid dysfunction (hyper-or hypothyroidism: 55-90% of cases) and in euthyroid individuals (3-8% of cases).

## Canonical autoAT

Anti-thyroid autoAbs of different levels of specificity which are dominated in a majority of patients with Graves' disease (70% and more) are known to be presented by canonical anti-TPO and anti-TG autoABs (60%) and, to a lesser extent, by anti-TGPO autoAbs (less 40%) to be canonical as well. Dynamics of anti-thyroid autoAbs would depend on the severity of the autoaggression and would result in the decrease prior to the early stages of the remission or in the burst at a time point preceding the exacerbation and/or the progression of the disease. Therefore, pre-early (*preclinical*) diagnostic protocol could identify and define the risks of Graves' disease to progress, and to thus implement the in-time therapeutic intervention to prevent the initiation of the pre-early (preclinical) stages of the disease in the overall irreversible pathologic process.

### Anti-TG autoAbs

Anti-TG autoAbs in Graves' patients were established to occur with a frequency of 50%.

### Anti-TPO autoAbs

High-rate occurrence and serum titers of anti-TPO autoAbs would illustrate common and featured complications and manifested forms of the disease (92-95% of cases), as well as subclinical Graves' disease (62-66%).

### Anti-TGRO autoAbs

In patients with Graves' disease highly-enriched populations of anti-TGPO autoAbs have been found.

### A phenomenon of clustering of thyroid autoAbs

The most common triplet found in patients with manifested forms of Graves’ disease is a serological panel of *anti-TG-anti-TPO-anti-TGPO* autoAbs. To lesser extent, the occurrence of a panel of the duplex *anti-TPO-anti-TGPO* autoAbs. No *anti-TG-anti-TGPO* duplexes have been identified in Graves' disease.

## Functionally active autoAT

In patients with Graves' disease featured by a high prevalence of functionally active autoAbs, in particular, the TSH-R and Ab-proteases have been dominated.

### TSH-R autoAbs

TSH-R autoAbs classified as functionally valuable Abs to mimic functions of the hormone and to trigger the development of the autoimmune disease by binding to the receptor and subsequent stimulation of the thyrocytes to be activated. One can distinguish TSH-stimulating Abs(TCA) to raise the production of thyroid hormones and TSH-blocking autoAbs (TBA), to block activation of the receptor and to be involved in the development of hypothyroidism and thyroid atrophy.

### AT-proteases

The prevalence of AT-proteases in patients with Graves' disease was reached to 77%. In this case, 60% of seropositive patients with AT-proteases belonged to a tandem consisting of the TG-and TPO-Ab-specific proteases simultaneously. In the early stages of Graves 'disease (with subclinical) TPO - specific antibody-protease in general are the only ones: they are 50% of patients with subclinical Graves' disease and 30% - with goiter degree 0 (in which the AT-protease least active). The activity of Ab-proteases increases during the destruction of the thyroid (hypothyroidism): from stage to stage of symptomatic complicated course (activity increased in two or more times). With a progression of the disease and the development of hyperthyroidism is also identified a tendency for the serological clustering to be raised.

## Modern serodiagnostic protocols in practice of endocrinologist s

Today, there are no defined serological criteria to allow for putting timely diagnosis of Graves' disease. One way of solving the problem is the use of functionally active Abs (including Ab-protease). A special role in solving the above-mentioned problems associated with the diagnosis of syndromal forms of the hyperthyroidism (in particular, in patients with thyroid functional autonomy) and/or with the preclinical serodiagnostic protocols to identify the preclinical stages of the illness and to thus predict the latter would belong to a family of Ab-proteases as highly specific molecular tools.

